# Assembly of a Tripeptide and Anti-Inflammatory Drugs into Supramolecular Hydrogels for Sustained Release

**DOI:** 10.3390/gels3030029

**Published:** 2017-08-03

**Authors:** Marina Kurbasic, Chiara D. Romano, Ana M. Garcia, Slavko Kralj, Silvia Marchesan

**Affiliations:** 1 Chemical & Pharmaceutical Sciences Department, University of Trieste, Via L. Giorgieri 1, 34127 Trieste, Italy; marinakurbasic.mk@gmail.com (M.K.); chiarad.romano@outlook.it (C.D.R.); anamariagarcia.1988@gmail.com (A.M.G.); slavko.kralj@ijs.si (S.K.); 2 Materials Synthesis Department, Jožef Stefan Institute, Jamova 39, 1000 Ljubljana, Slovenia

**Keywords:** peptides, d-amino acids, chirality, hydrogels, drugs, release, self-assembly, co-assembly, naproxen, ketoprofen

## Abstract

Supramolecular hydrogels offer interesting opportunities for co-assembly with drugs towards sustained release over time, which could be achieved given that the drug participates in the hydrogel nanostructure, and it is not simply physically entrapped within the gel matrix. ^d^Leu-Phe-Phe is an attractive building block of biomaterials in light of the peptide’s inherent biocompatibility and biodegradability. This study evaluates the assembly of the tripeptide in the presence of either of the anti-inflammatory drugs ketoprofen or naproxen at levels analogous to commercial gel formulations. Fourier-transformed infrared (FT-IR), circular dichroism, Thioflavin T fluorescence, transmission electron microscopy (TEM), and oscillatory rheometry are used. Drug release over time is monitored by means of reverse-phase high performance liquid chromatography, and shows different kinetics for the two drugs.

## 1. Introduction

Supramolecular hydrogels, and especially those formed by short peptides, have attracted increasing interest as innovative vehicles for drug loading and release, especially if sustained or *on demand* [1]. Their obvious advantages over other classes of gelators include ease of preparation, low cost, fine control over molecular composition, inherent biocompatibility, biodegradability, and possibility for biomimicry [2]. Such advantages become more prominent in the case of minimalist systems composed of up to three amino acids that can easily be prepared also by liquid-phase methods [3]. Tripeptides composed of both d- and l-amino acids are particularly attractive, as they may allow for specific peptide conformation, increased resistance towards protease degradation, and at times even unexpected bioactivity, relative to their homochiral L-counterparts [4,5,6,7]. Other approaches to increase gelator resistance to enzymatic hydrolysis include the use of *N*-aromatic derivatization [8,9,10], and can be combined with other approaches, such as the use of dehydro (∆) amino acids [11,12].

Traditionally, drugs have been physically entrapped in a polymer matrix; however, an initial burst is typically observed, and generally the release occurs rapidly if the drug simply diffuses out of the matrix [13]. Sustained delivery can occur by design, e.g., through the engineering of hydrogel nanopore size, through the use of microparticles, or if the drug is engaged with the hydrogel matrix, i.e., through non-covalent or covalent bonds that can be hydrolysed [14]. A recent approach is co-assembly, whereby the drug participates with a gelator in the supramolecular nanostructured gel [15]. In this case, release happens slowly as the nanostructures disassemble. However, for co-assembly to occur, a number of physico-chemical requirements need to be met, of which only a few can be anticipated, since it is not yet exactly known how gelator molecules behave through the self-assembly process, and to what extent can they tolerate other molecules (e.g., drug cargo). Naproxen and other drugs were effectively engaged through hydrogen bonding in a supramolecular hydrogel [16]. The neurotransmitter γ-amino isobutyrric acid (GABA) was proposed to bridge non-covalently between Fmoc-lysine molecules of a supramolecular hydrogel [17].

In the realm of self-assembled, ultrashort (≤4 amino acids) peptide hydrogels, several examples have been reported for drug loading and release, simply based on physical entrapment [18,19,20,21]. Drug covalent conjugation was also shown to be possible and to not compromise self-assembly [22], for instance for 5-fluorouracil Fmoc-Lys-Lys conjugates [23]. The phosphorylated d-peptide derivative Nap-^d^Phe-^d^Phe-^d^Lys-^d^Tyr(P) is a convenient precursor of the hydrogelator Nap-^d^Phe-^d^Phe-^d^Lys-^d^Tyr, since it can be enzymatically dephosphorylated by a phosphatase, but it is resistant toward protease-mediated hydrolysis. The compound presents a primary amine on the side chain of lysine that can be used to bind drugs, such as an anti-HIV prodrug [24] or taxol [25]. Taxol itself was used as self-assembling motif, bound through a linker to the reduced form of the glutathione tripeptide [26]. Naproxen is yet another drug that was used as a self-assembling template bound to ^d^Phe-^d^Phe, ^d^Phe-^d^Phe-^d^Lys, or ^d^Phe-^d^Phe-^d^Lys-^d^Tyr thanks to its aromatic character and rigid, hydrophobic structure [27].

**Figure 1:**
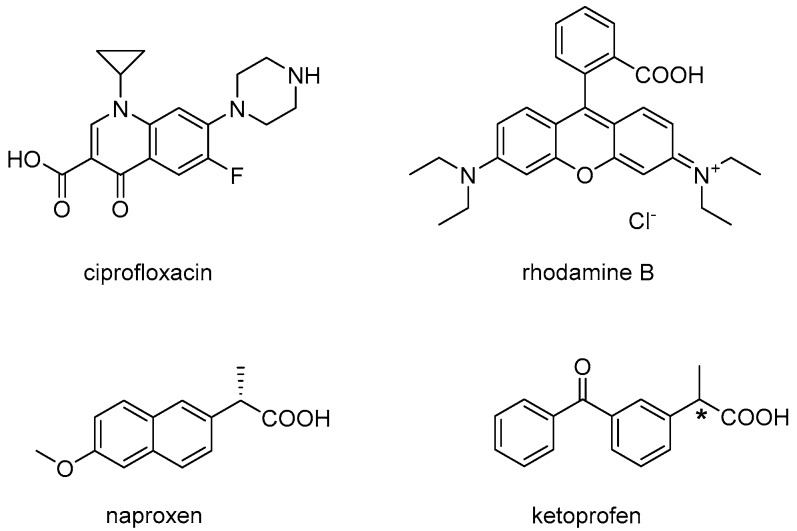
Chemical structures of drug model compounds evaluated for co-assembly with the peptide ^d^Leu-Phe-Phe in previous studies (**top**) and this study (**bottom**). * denotes the chiral centre of the racemic mixture that composes ketoprofen.

In line with principle, it should also be possible to engage drugs in non-covalent interactions, and thus co-assembly with an ultrashort peptide, so that the release kinetics would be determined by nanostructure disassembly. However, considering the small molecular weight of ultrashort peptides (<1 kDa), it is apparent how their co-assembly with a drug would be heavily influenced by the drug, and thus be challenging to fine-tune. Indeed, to our knowledge, there is only one reported example of co-assembly between a drug and an (unprotected) tripeptide gelator resulting in a markedly different nanostructure morphology relative to the peptide alone, where drug–peptide interaction was established by spectroscopy [15]. In this case, ^d^Leu-Phe-Phe was dissolved with hydrophobic ciprofloxacin at alkaline pH and it gelled upon a pH trigger to neutral. It was proposed that π–π interactions between the two hydrophobic, aromatic compounds played a key role in co-assembly, although other interactions of ionic nature or hydrogen bonding could not be excluded. Importantly, a sustained drug release was achieved, with just over 40% of the drug being delivered over 6 days [15]. ^d^Leu-Phe-Phe was shown to co-assemble into hydrogels of different nanostructure also with rhodamine dye, and similarly to the case above, the spectroscopic signature, nanomorphology, and viscoelastic properties were affected [28]. In both studies, the small molecules displayed an aromatic core and both amine and carboxylic acid functional groups (Figure 1, top), which may play a role in establishing non-covalent interactions with the peptide during assembly.

In an attempt to shed further light on the interesting phenomenon of co-assembly for sustained drug delivery, we thus chose for this study the anti-inflammatory drugs naproxen and ketoprofen (Figure 1, bottom), which have a very similar structure and hydrophobicity (i.e., an octanol-water partition coefficient, logKow, of 3.18 and 3, respectively) [29]. This work would allow investigators to evaluate whether an ionizable carboxylic group and an aromatic core are indeed sufficient to allow co-assembly with the peptide, and whether the conjugated π-system of the naphthalene unit of naproxen displays a net advantage, relative to the two benzene rings (not fused together) of ketoprofen, to interact with the tripeptide. Another aspect that could be assessed by comparing these two molecules is the importance of the stereoconfiguration at the chiral centre, being naproxen enantiomerically pure, while ketoprofen a racemic mixture. Finally, both drugs are formulated as hydrogels in commercial products, and thus they constitute a model of potential relevance in terms of the future application of peptide hydrogels as drug delivery vehicles. Therefore, this work evaluates the assembly of ^d^Leu-Phe-Phe into hydrogels in the presence of either naproxen or ketoprofen, at the same loading level as commercial products, towards sustained drug release.

## 2. Results and Discussion

The tripeptide ^d^Leu-Phe-Phe is an established gelator in phosphate buffer at neutral pH. In a typical protocol, it is first dissolved at pH 12 thanks to repulsion between the negatively charged peptide molecules, then it rapidly self-assembles as the pH is lowered to neutral and the peptide becomes zwitterionic [15]. Ketoprofen or naproxen were thus dissolved in the alkaline buffer as sodium salts, and were mixed with the peptide prior to assembly to favour their subsequent incorporation into the supramolecular structure. In order to assess the potential applicability of the resulting soft materials as drug delivery vehicles, each drug was loaded at the level typically found in commercial formulations, i.e., 25 and 100 mg/mL, respectively.

### 2.1. Hydrogel Characterisation

**Figure 2:**
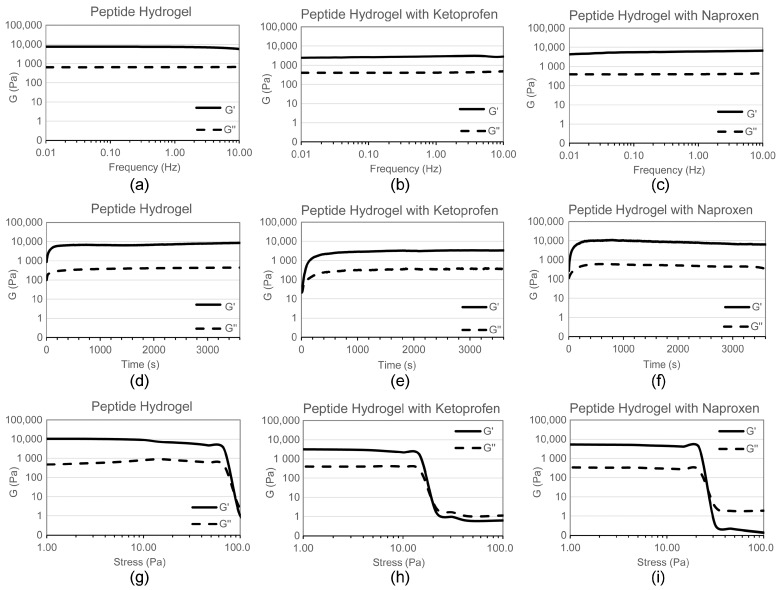
Oscillatory rheometry analysis of hydrogels (**a**–**c**) frequency sweeps; (**d**–**f**) time sweeps; (**g**–**i**) stress sweeps.

Self-supporting hydrogels were obtained in both cases, as confirmed by oscillatory rheometry (Figure 2). The elastic modulus G′ of the peptide hydrogel was significantly lowered upon the inclusion of ketoprofen, from 9 to 3 kPa, indicating the formation of a softer hydrogel. Interestingly, a minor difference in G′ was observed upon the inclusion of naproxen (G′ = 6 kPa), despite this drug being loaded at a fourfold higher level. The kinetics of assembly were not affected by the addition of either drug, while the resistance to applied stress was notably reduced. Indeed, the viscoelastic moduli of the peptide hydrogel maintained linearity up to 60 Pa, which was reduced to 15 Pa upon the addition of either drug. This rheological behavior overall was thus significantly different when compared to ^d^Leu-Phe-Phe hydrogels co-assembled with either ciprofloxacin [15] or rhodamine [28]. In such cases, the elastic modulus G′ was also lowered: we can hypothesise that in all cases, the presence of cargo disrupts the bundling of fibrils into thicker fibers as it happens for the peptide alone, possibly because of an altered fibril surface topography. However, in both of the literature examples [15,28], hydrogel resistance to applied stress was increased upon the inclusion of cargo, suggesting that the presence of either a drug or dye effectively increased the interconnectivity of the supramolecular fiber network (as confirmed by TEM). By contrast, the soft materials of this study containing either naproxen or ketoprofen likely display fewer connection points between fibrils relative to the peptide alone, thus the lowered resistance to applied stress.

### 2.2. Nanostructure Morphology

**Figure 3:**
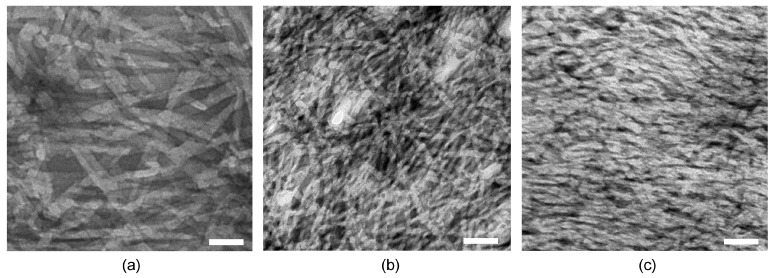
TEM micrographs of (**a**) peptide hydrogel, (**b**) peptide hydrogel with ketoprofen, and (**c**) peptide hydrogel with naproxen. Scale bar = 50 nm.

Transmission electron microscopy (TEM) was thus employed to evaluate differences in nanomorphology that could explain the different viscoelastic properties discussed above (Figure 3). In all cases, a dense network of fibers was observed. Individual fibrils displayed a narrow diameter distribution, with minor, but statistically significant, differences between samples with or without either drug. In fact, fibrils formed by the peptide alone were 9.8 ± 1.4 nm wide, and became thinner upon the inclusion of either drug (i.e., 7.3 ± 1.0 nm for the peptide with ketoprofen, and 8.3 ± 2.2 nm for the peptide with naproxen). Importantly, and only in the case of peptide alone, fibrils intertwined into bundles as thick as 30 nm, which could provide branching points, thus increasing the resistance of the hydrogel network to applied stress, in agreement with the rheometric data. Thicker bundles could also explain the higher stiffness registered for the peptide hydrogel.

It is interesting to compare the TEM micrographs of these systems relative to those in the literature, where ^d^Leu-Phe-Phe is assembled in the presence of other components. Nanomorphological behaviour is strikingly similar to the case of both hydrophilic rhodamine dye [28] or carbon nanodots (displaying carboxylic acid and amine groups on their surface) [30] that were dissolved/dispersed with the peptide in the alkaline buffer. By contrast, when ^d^Leu-Phe-Phe was co-assembled with hydrophobic ciprofloxacin, the fibers’ nanomorphology was markedly different, showing nanospheres attached to the fibrils, and fibrils with a notably increased level of branching relative to the peptide alone [15]. We propose that, as suggested by the rheology data, the most likely scenario, in the presence of other components dissolved with the peptide prior to assembly, involves peptide stacking in a similar fashion as when it is alone, although the second component likely changes fibril surface topography and thus hampers bundling into thicker fibers, lowering the elastic modulus. Instead, a significantly different nanomorphology was achieved only in the case of hydrophobic ciprofloxacin, suggesting this drug has the required properties to engage at a deeper level in the assembly process with the peptide.

### 2.3. Spectroscopic Study of Peptide Conformation

**Figure 4:**
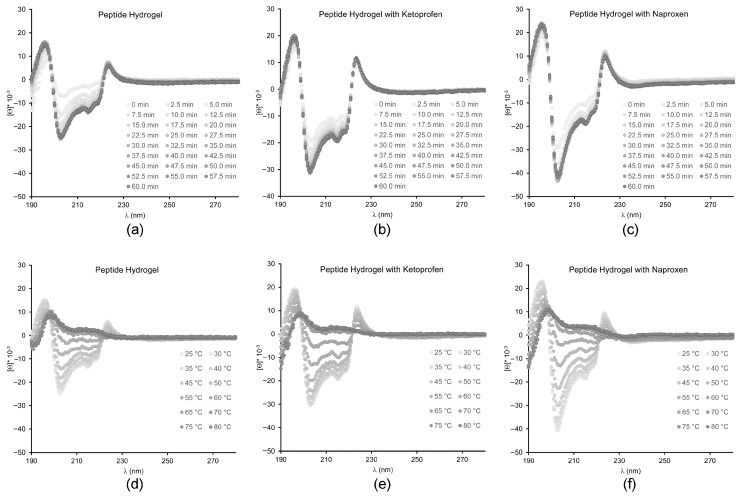
CD spectra of hydrogels; (**a**–**c**) kinetics over 60 min, (**d**–**f**) heating ramps up to 80 °C.

Circular dichroism (CD) spectroscopy was employed to assess changes in peptide conformation upon assembly in the presence of either drug (Figure 4). Overall, the kinetics of assembly were not significantly altered by either drug’s inclusion in the system, in agreement with the rheometric data. The main features of the characteristic CD signature of the peptide in its supramolecular state were also maintained in all cases, although quantitative effects were observed. In particular, the negative signal for the amide bond in the 200–220 nm region that corresponds to the peptide conformation was more intense upon the addition of ketoprofen, although the relative intensities of the negative peaks at 205, 216 and 219 nm were preserved. Instead, all peaks were more intense upon the addition of naproxen, and also their relative intensities were different, with the minimum at 205 nm being the most affected, and reaching nearly twofold intensity. This data suggests that the peptide backbone did not significantly change conformation upon the inclusion of ketoprofen, although the surrounding chiral environment was slightly altered. Assembly in the presence of naproxen led to more significant changes, suggesting variations also in peptide conformation. Heating ramps up to 80 °C instead indicated that all materials displayed analogous thermoresistance, with the supramolecular signal being completely lost at 60 °C in all cases. Therefore, despite the observed differences in the peptide chiral environment, overall drug inclusion did not compromise the thermostability of the supramolecular assemblies.

If we compare the CD data with the literature, we can see that the most similar case is the one of ^d^Leu-Phe-Phe assembled in the presence of carbon nanodots [30], whereby the 1–2 nm-wide components altered quantitatively, but not qualitatively, the CD signal in the 200–220 nm region. However, in that case, the minimum at 205 nm was reduced, and the others at 216 and 219 nm were increased. This data suggests that CD is extremely sensitive at revealing changes in the chiral environment of the peptide, which can be affected in different ways depending on the chemical nature of the other components present in the system. Very different was the case of rhodamine dye [28] or ciprofloxacin [15] that altered also qualitatively the peptide CD signature in the 200–220 nm region, suggesting more interactions with the peptide, and thus affecting the local environment of the peptide amide bonds.

**Figure 5:**
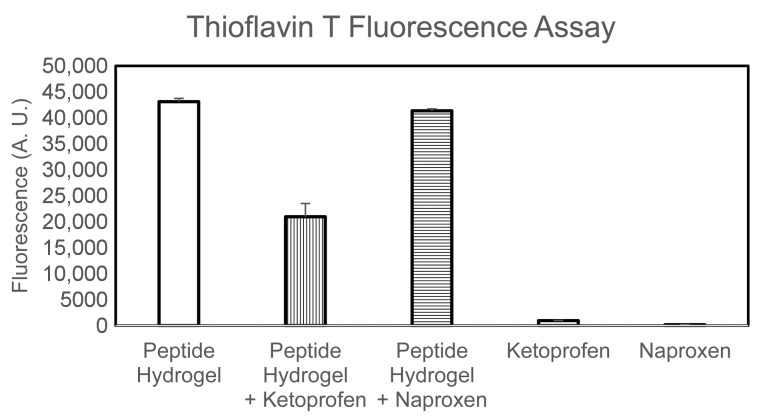
Thioflavin T fluorescence assay.

Fourier-Transformed Infrared (FT-IR) spectroscopy was then used to verify any significant difference in the peptide signal in the amide I region that may indicate altered peptide conformation during assembly in the presence of either drug. However, it should be noted that the drugs were present at notably higher amounts relative to the peptide (i.e., sixfold in weight for ketoprofen, and 24-fold in weight for naproxen), therefore the drug signal completely dominated the IR spectrum (see Figure S1). Thioflavin T fluorescence was next used to evaluate changes in the amyloid supramolecular structure (Figure 5). The dye is widely used to quantify amyloid presence, since it is known to laterally bind to the hydrophobic grooves of amyloid fibrils composed of at least four-consecutive beta-sheets [31]. Upon binding, the reduced rotation of the single bond that connects the two aromatic units composing the dye leads to marked fluorescence, which is not observed in the absence of amyloids [32]. Fluorescence intensity is thus used to quantify the number of amyloid fibrils present in a sample. Indeed, controls containing either drug did not display any fluorescence at all. By contrast, all hydrogel samples displayed marked fluorescence upon Thioflavin T binding. Interestingly, the samples containing naproxen displayed only a 4% reduction in fluorescence relative to the peptide alone. The samples containing ketoprofen displayed a notable 51% reduction of the signal. This data suggests that naproxen inclusion in the system does not compromise amyloid assembly, while systems with ketoprofen likely display significantly less amyloid surface area available for Thioflavin T binding, which could be the result of a lower number of fibrils or an altered fibril surface. It is worth noting that naproxen is enantiomerically pure, while ketoprofen is a racemic mixture, thus one enantiomer may engage less favourably in interactions with the chiral peptide.

### 2.4. Drug Release Studies

**Figure 6:**
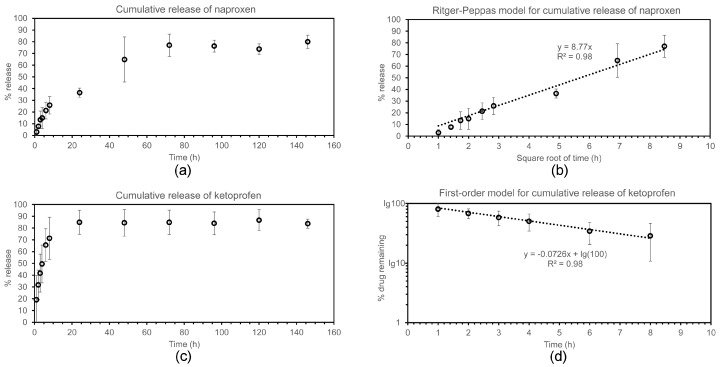
Drug release study for naproxen (**a**,**b**) and ketoprofen (**c**,**d**).

Drug release was monitored over a 6-day period (Figure 6) at physiological conditions (i.e., phosphate-buffered saline solutions at 37 °C, with mild shaking), by using high-performance liquid chromatography (HPLC). Ketoprofen sodium salt has a water solubility at pH 7 corresponding to approximately 600 mg/mL [33], while naproxen sodium salt has a water solubility of 334 mg/mL at 37 °C [34]. Therefore, despite the high loading of the hydrogel formulations described in this work, both drugs were well below their solubility limit, and the release experiments were carried out in sink conditions.

In the case of naproxen (Figure 6a,b), the majority (i.e., 77.0 ± 9.5%) of the drug was released within the first 72 h, with no significant further release at later timepoints. The first 72 h-release kinetics followed neither zero nor first order equations. During this time, the cumulative % drug release displayed linearity with the square root of time, as described with the empirical equation of Ritger–Peppas as applied to Fickian one-dimensional diffusion for the initial 60% of total released drug [35]. This empirical model can be applied to a variety of drug formulation geometries, and both non-swelling or moderately-swelling matrices, as long as sink conditions are maintained [36]. In terms of geometry, the hydrogels analysed in this work adhered to the bottom of the vials, so that release effectively occurred only in the vertical direction through the hydrogels’ top surface onto the medium above. Sink conditions were maintained throughout the experiment as mentioned above. It should also be noted that the tripeptide hydrogels described in this work did not undergo visible swelling, which is not expected considering that they were formed from phosphate-buffered solutions and phosphate buffer is also the release medium. It is also interesting to note that, from a purely mathematical point of view, the linear relationship observed between cumulative drug release and the square root of time is also found in the Higuchi model. However, the underlying theoretical assumptions of the Higuchi model are not valid for this work, and thus its application in this context would be inappropriate [37].

In the case of ketoprofen (Figure 6c,d), despite this drug being loaded at one-quarter in weight relative to the case of naproxen described above, the release followed much faster kinetics, suggesting less interaction with the hydrogel matrix. Indeed, drug release was completed during the first 24 h, after which time further release was negligible. Interestingly, in this case, the best fit for the release of up to 70% of the drug followed first-order kinetics, as typically observed for matrix-type devices [38]. Despite the drug being present as a racemic mixture, no two-step process was observed to suggest a different level of interaction between each enantiomer and peptide. Therefore, we can conclude that the drug interaction, observed by the spectroscopic, microscopic, and mechanical means described above, is not very specific and likely plays a minor role in the release process.

## 3. Conclusions

Rational design towards the co-assembly of small molecule drugs and a simple, unprotected tripeptide into supramolecular hydrogels for sustained release poses many challenges. In this work, we demonstrated that it is possible to assemble a tripeptide into a hydrogel at physiological conditions with an amount of drug corresponding to commercial formulations, both in the case of ketoprofen and naproxen. The drugs were not just physically entrapped in the hydrogel matrix, since significant changes relative to the peptide hydrogel without drugs were observed by oscillatory rheometry, TEM, CD, FT-IR, and Thioflavin T fluorescence. Ketoprofen appeared to have a major impact on the ability of the peptide to form thicker bundles of fibrils, as reflected by Thioflavin T fluorescence and rheology. Naproxen appeared to have an impact also on peptide conformation, whilst the fibrils were slightly thicker relative to ketoprofen, and overall the amyloid character and the hydrogel stiffness were more similar to the peptide hydrogel without a drug.

Drug release studies highlighted differences between the two materials, since they did not follow analogous kinetics. Remarkably, despite naproxen being loaded at a fourfold higher level relative to ketoprofen, the naproxen-containing hydrogel showed slower release kinetics that fitted the Ritger–Peppas empirical model for the initial 70% of the drug release over 3 days. By contrast, ketoprofen followed more typical first-order kinetics, and overall drug delivery was more rapid as it mainly occurred during the first 24 h. It is highly possible that the naphthalene unit of naproxen is more efficient than the benzene rings of ketoprofen at engaging in non-covalent interactions with the aromatic units of the peptide. Indeed, the phenylalanine residues of ^d^Leu-Phe-Phe were hypothesised to form hydrophobic zippers [39], also found in other short peptide systems [40], and generally in amyloids [41]. It is well-established that naproxen covalently bound to peptides plays an adjuvant role in self-assembly driven by hydrophobic interactions in water [27], as well as similar derivatives of the naphthalene unit [9,42,43]. If we consider the high level of conjugation displayed by the other small molecules able to engage with ^d^Leu-Phe-Phe in supramolecular structures (Figure 1), we can conclude that aromatic rings not fused together, as in the case of ketoprofen, are unlikely to be as successful for co-assembly to occur.

## 4. Materials and Methods

### 4.1. Materials

The 2-chlorotrytil resin, *O*-Benzotriazole-*N*,*N*,*N*,*N*′-tetramethyl-uronium-hexafluoro-phosphate (HBTU), and Fmoc protected *N*-trityl-l-histidine and d-phenylalanine were purchased from GL Biochem (Shanghai) Ltd. (Shanghai, China). All solvents were purchased of analytical grade from Merck (Milan, Italy). Drugs and chemicals were from Sigma (Milan, Italy). High purity Milli-Q-water (MQ water) with a resistivity greater than 18 MΩ·cm was obtained from an in-line Millipore RiOs/Origin system (Merck, Milan, Italy). The ^1^H-NMR spectra were recorded at 400 MHz, and the ^13^C-NMR spectra were recorded at 100 MHz on a Varian Innova Instrument (Agilent Technologies, Milan, Italy) with chemical shift reported as ppm (in dimethyl sulfoxide (DMSO) with tetramethylsilane as internal standard). ESI-MS spectra were recorded on an Infinity 6120 single quadrupole LC-MS system (Agilent Technologies, Milan, Italy).

### 4.2. Peptide Synthesis and Purification

The peptide was synthesised using standard Fmoc solid phase peptide synthesis with HBTU activation. Briefly, Fmoc-amino acid deprotection was performed in a sintered funnel, with continuous stirring, in 20% piperidine in *N*,*N*-dimethylformamide (DMF) for 20 min (2 × 10 min) until both bromophenol blue and acetaldehyde/chloranil tests were positive. HBTU activation was performed with 2.5 equiv. of Fmoc-amino acid, 2.0 equiv. of HBTU, and 2.0 equiv. of HOAt in DMF (4 mL for every equiv. of resin), with *N,N*-diisopropyl ethyl amine (DIPEA, 2 mL of a 1 M solution in DMF for every equiv. of resin). Coupling was performed at room temperature for 1.5 h in a sintered funnel with continuous stirring, and completeness was monitored by both bromophenol blue and acetaldehyde/chloranil tests after thorough washes with DMF and DCM (Dichloromethane). The final cleavage was obtained using a mixture of TFA/DCM/TIPS/water (47.5:47.5:2.5:2.5) (TFA: trifluoroacetic acid; TIPS: triisopropyl silane). The crude peptide was too hydrophobic to be precipitated in cold ether, thus the majority of TFA was evaporated under argon flow, and the remaining oil was dissolved in a mixture of acetonitrile/water and then purified by reverse-phase HPLC (Agilent Technologies, Santa Clara, CA, USA). The HPLC Agilent 1260 Infinity system was equipped with a preparative gradient pump (1311B), semipreparative C-18 column (Kinetex, 5 microns, 100 Å, 250 mm × 10 mm, Phenomenex, Torrance, CA, USA), autosampler (G1329B), and Photodiode Array detector (G1315C). The gradient used consisted of acetonitrile (MeCN)/water with 0.1% TFA with the following program: *t* = 0–2 min, 25% MeCN; *t* = 12 min, 50% MeCN; *t* = 15 min, 95% MeCN; *t* = 16 min, 95% MeCN (Retention time *t*_R_ = 6.8 min). The compound was then freeze-dried to yield the corresponding peptide as a white fluffy powder. Peptide identity was verified by ESI-MS, ^1^H-NMR, and ^13^C-NMR [39].

### 4.3. Hydrogel Preparation

The peptide was dissolved in sodium phosphate buffer (0.1 M pH 11.8), and then diluted with an equal volume of sodium phosphate at acidic pH (0.1 M pH 5.8) to a final peptide concentration of 10 mM, obtaining a final pH of 7.2 ± 0.1. All buffer solutions were filtered (0.2 μm) prior to use. In the case of the naproxen-containing hydrogels, in a typical protocol naproxene was dissolved with 200 μL of 1 M NaOH and 150 μL sodium phosphate buffer (0.1 M pH 11.8), then the peptide ^d^Leu-Phe-Phe was added to achieve a final concentration of 10 mM of peptide, with another 150 μL of sodium phosphate buffer (0.1 M pH 5.8) to obtain a final pH of 7.1 ± 0.1. All buffer solutions were filtered (0.2 μm) prior to use. The final concentration of naproxene was 100 mg/mL. In the case of the ketoprofen-containing hydrogels, ketoprofen was dissolved with 50 μL of 1 M NaOH and 200 μL of sodium phosphate buffer (0.1 M pH 11.8), then the peptide ^d^Leu-Phe-Phe was added to a final concentration of 10 mM of peptide, with another 250 μL of sodium phosphate buffer (0.1 M pH 5.8) to obtain a final pH of 7.1 ± 0.1. All buffer solutions were filtered (0.2 μm) prior to use. The final concentration of ketoprofen was 25 mg/mL.

### 4.4. Oscillatory Rheometry

Dynamic time sweep rheological analysis was conducted on a Malvern Kinexus Ultra Plus Rheometer (Alfatest, Milan, Italy) with a 20 mm stainless steel parallel plate geometry. The temperature was maintained at 25 °C using a Peltier temperature controller (Alfatest, Milan, Italy). Samples were prepared in situ and immediately analysed with a gap of 1.00 mm. Time sweeps were recorded for 1 h, using a frequency of 1.00 Hz and a controlled stress of 2.00 Pa. After 1 h, frequency sweeps were recorded using a controlled stress of 2.00 Pa, and then stress sweeps were recorded using a frequency of 1 Hz.

### 4.5. Transmission Electron Microscopy

TEM micrographs were acquired on a Jeol, JEM 2100 (Tokyo, Japan) at 100 kV. TEM grids (copper-grid-supported lacey carbon film) were first exposed to UV-ozone cleaner (UV-Ozone Procleaner Plus, BioForce Nanosciences, Ames, IA, USA) for 45 min to make the grid surface more hydrophilic. Then, 24 h-aged gels were precisely deposited on a TEM grid, dried for 15 min at room temperature and contrasted by aqueous tungsten phosphate solution (pH 7.4). The average size of the nanostructures was determined by taking into account at least 100 measurements.

### 4.6. Circular Dichroism

A 0.1 mm quartz cell was used on a Jasco J-815 Spectropolarimeter (Jasco Europe, Cremella, Italy), with 1 s integrations, one accumulation, and a step size of 1 nm with a bandwidth of 1 nm over a range of wavelengths from 190 to 280 nm at 25 °C unless otherwise stated. Samples were freshly prepared directly in the CD cell and the spectra immediately recorded.

### 4.7. Fourier-Transformed Infrared Spectroscopy

FT-IR spectra were registered using the KBr-pellet method. The gel was prepared, and after 24 h it was dried under vacuum. Then, it was mixed with KBr to make the pellet. Spectra were acquired at 1 cm^−1^ resolution and 128 accumulations on a Perkin Elmer System 2000 (Perkin Elmer, Milan, Italy).

### 4.8. Thioflavin T Fluorescence Assay

Gels (150 μL) were prepared directly inside the wells of Greiner 96 U Bottom Black Polystyrene plates (VWR, Milan, Italy). After 1 h, 30 μL of a solution of Thioflavin T (22.2 M in 20 mM glycine-NaOH pH 7.5, filtered with a 0.2 μm filter) were added into the wells. After 15 min, the fluorescence emission was analysed using an Infinite M1000 pro (Tecan, Milan, Italy), selecting an excitation wavelength of 446 nm and an emission wavelength of 490 nm, with a bandwidth of 20 nm. Each condition was repeated twice in triplicate. The average and standard deviations were calculated and plotted with Excel.

### 4.9. Drug Release Assay

Hydrogels were prepared as described above (0.500 mL total volume) in an amber glass vial, and were left to self-assemble overnight. The following morning, 8 mL of phosphate-buffered saline (PBS) were added on top, filling the vials. The vials were incubated at 37 °C, 60 rpm, and at various timepoints. Fifty microliter (50 μL) samples were taken and added to 0.200 mL of water and analysed by reverse-phase HPLC (absorbance at 254 nm, same program as described above for peptide purification). Each experiment was repeated three times. The average and standard deviation values were calculated and plotted with Excel.

## Supplementary Materials

Supplementary materials can be found at www.mdpi.com/1422-0067/3/3/29/s1. Figure S1: Amide region of FT-IR spectra of drugs alone (**a**,**b**) and included in the peptide hydrogels (**c**,**d**).
