# Supplementary material for: Assembly of a Tripeptide and Anti-Inflammatory Drugs into Supramolecular Hydrogels for Sustained Release
Source: Gels. 2017 Aug 3;3(3):29. doi: 10.3390/gels3030029 (PMC6318665; doi:10.3390/gels3030029)
Supplement: Supplementary file 1 [file gels-03-00029-s001.pdf]

Supplementary Information

Assembly of a tripeptide and anti-inflammatory drugs into supramolecular hydrogels for sustained release

Marina Kurbasic [1,‡], Chiara D. Romano [1,‡], Ana M. Garcia [1], Slavko Kralj [1,2] and Silvia Marchesan [1,*]

[1] Chem. Pharm. Sc. Dept., University of Trieste; Via L. Giorgieri 1, Trieste 34127, Italy, smarchesan@units.it
[2] Materials Synthesis Dept., Jožef Stefan Institute, Jamova 39, 1000 Ljubljana, Slovenia
‡ These authors contributed equally to the work
* Correspondence: smarchesan@units.it; Tel.: +39-040-558-3923

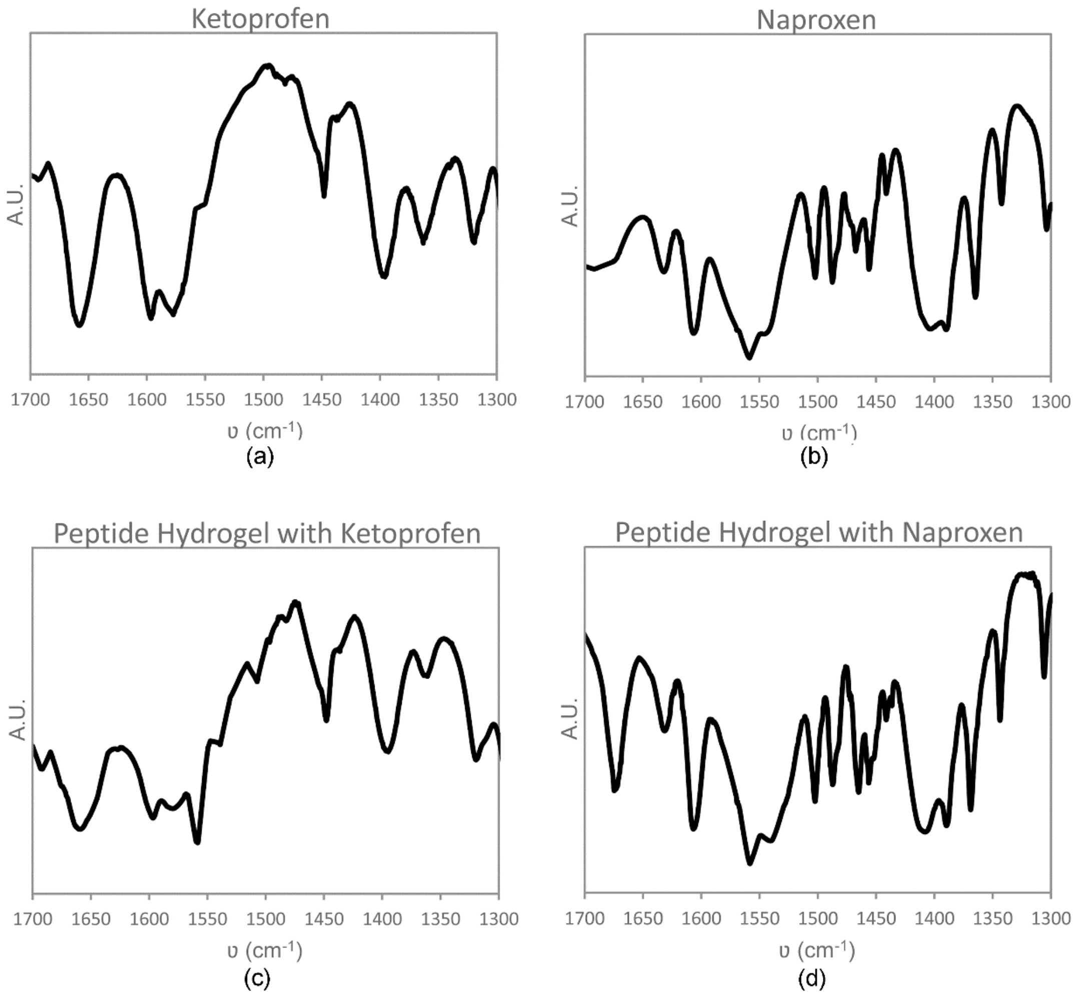

Figure S1. Amide region of FT-IR spectra of drugs alone (**a-b**) and included in the peptide hydrogels (**c-d**).
